# Recent Advances in Studies of Serum Amyloid A: Implications in Inflammation, Immunity and Tumor Metastasis

**DOI:** 10.3390/ijms26030987

**Published:** 2025-01-24

**Authors:** Yixin Chang, Yezhou Liu, Yuanrui Zou, Richard D. Ye

**Affiliations:** 1 Kobilka Institute of Innovative Drug Discovery, School of Medicine, The Chinese University of Hong Kong, Shenzhen 518172, China; 2 The Chinese University of Hong Kong, Shenzhen Futian Biomedical Innovation R&D Center, Shenzhen 518000, China

**Keywords:** serum amyloid A, acute-phase response, inflammation, tumor microenvironment, innate immunity, mouse models

## Abstract

Research on serum amyloid A (SAA) has seen major advancement in recent years with combined approaches of structural analysis and genetically altered mice. Initially identified as an acute-phase reactant, SAA is now recognized as a major player in host defense, inflammation, lipid metabolism and tumor metastasis. SAA binding and the neutralization of LPS attenuate sepsis in mouse models. SAA also displays immunomodulatory functions in Th17 differentiation and macrophage polarization, contributing to a pro-metastatic tumor microenvironment. In spite of the progress, the regulatory mechanisms for these diverse functions of SAA remain unclear. This review provides a brief summary of recent advances in SAA research on immunity, inflammation, tumor microenvironment and in vivo models.

## 1. Introduction

Serum amyloid A (SAA) is an acute-phase reactant, a precursor of amyloid A (AA) and an apolipoprotein of HDL [1]. These definitions reflect our understanding of SAA at different stages of its discovery. The inferred functions of SAA have attracted attention of both clinicians and bench scientists, resulting in nearly 10,000 publications based on PubMed search using SAA or serum amyloid A as keyword in titles and abstracts. In healthy individuals, SAA presents at a low level in the serum. Within the first two days of an acute-phase response, the serum level of SAA rises markedly by up to 1000-fold [2]. Acute-phase response can be induced by drastic changes to the host including trauma, infection, inflammation and malignancy [3]. Hence, SAA has been widely characterized as a biomarker of inflammation and associated clinical disorders that reflect the onset, severity and prognosis of diseases [4,5,6]. In the past quarter century, research on SAA has led to a positive correlation of acute-phase SAA level with the expression of proinflammatory cytokines, along with the discovery of receptors that could mediate the proinflammatory activities of SAA (summarized in [7]). However, in other published studies, SAA was found to have anti-inflammatory activities [8,9,10]. These activities may result from established antibacterial functions of SAA including the binding of outer membrane protein A [11], opsonizing for phagocytosis [12], and the neutralization of major bacterial toxins such as LPS [13]. In doing so, SAA may help to promote the resolution of infection-associated inflammatory response. Research on SAA has been hampered by the lack of reagent standard, such that a widely used recombinant SAA by *E. coli* expression was suspected to have bacterial contaminants that could alter the binding to pro-/anti-inflammatory receptors and subsequent physiological outcomes [14]. Moreover, the use of a recombinant SAA with substituted amino acids might have caused an exaggerated effect in certain in vitro studies [15]. These reports cast doubts on the observed proinflammatory activities of SAA despite the continued use of bacteria-produced recombinant SAA by researchers [16,17].

In the past decade, technical advancement has propelled SAA research and provided answers to some of the above questions. Structural studies have shown that SAA can exist in monomer, dimers and hexamers [18,19]. The presence of SAA in these higher order forms suggests that the quaternary structure of SAA and its transformation may explain some paradoxical observations reported in the literature. Another major advance in SAA research is the increased use of knockout and transgenic mice for studies of SAA functions related to human diseases. To date, there are nearly 30 published studies of genetically altered mice with an individual or combined knockout of Saa genes and a forced expression of human *SAA1* as a transgene. The genetic approaches have effectively overcome the bacterial contamination problem associated with the use of *E. coli*-derived recombinant SAA preparations. Of interest, several studies employed both genetically altered mice and in vitro verification with *E. coli*-derived recombinant SAA proteins, and the results consistently demonstrate the functions of SAA in Th17 differentiation [16,17], TLR2 usage and influence to tumor microenvironments [20,21,22]. As discussed below, these genetically modified mice have been successfully used in disease models including inflammatory bowel disease, acute lung injury, liver cancer and Alzheimer’s disease (AD), providing useful information for the future development of SAA-based therapeutics.

There have been recent reviews on SAA as an acute-phase protein [23,24] and an inflammatory biomarker [6], as well as on the structural properties of SAA [25] and the involvement of SAA in disease development and progression [26,27,28]. There were also reviews published in the past on SAA isoforms and the SAA receptors [7,29]. In view of the published literature, the present review is mainly focused on the recent development of SAA studies within the past 8–10 years with an emphasis on the correlation of SAA structures and its in vivo functions.

## 2. Association of SAA Expression with Major Diseases

The term “acute-phase SAA” refers to isotypes of SAA whose expression is rapidly and markedly induced during acute-phase response [1,3]. These SAA isotypes include SAA1 and SAA2 in humans and Saa1, Saa2 and Saa3 in mice. There is also an SAA isotype, SAA4, in humans and mice that is constitutively expressed and receives less attention [30,31,32]. A close association has been identified between an elevated expression level of SAA and the progression of several diseases.

### 2.1. SAA as a Disease Biomarker

The majority of the published literature focuses on the association of elevated SAA expression level and diseases. Such an association places acute-phase SAA as a biomarker for infectious and inflammatory diseases, cancer and metabolic diseases, as briefly summarized in Table 1 below. Other than AA amyloidosis, there are very few cases in which SAA has been identified as a cause for the disease. For example, SAA polymorphism has been reported to contribute to the development of nasopharyngeal cancer [33].

### 2.2. Regulation of SAA Gene Expression

The expression of acute-phase SAA is regulated mainly at the transcriptional level. The promoters of several inducible SAA isotypes, including human *SAA1* and *SAA2*, contain binding sites for transcription factors including NF-κB, C/EBP, YY1, AP-2, SAF, Sp1 and STAT3 [2,64,65]. As a result, the transcript of acute-phase SAA is rapidly induced by inflammatory factors such as TNFα, IL-1β and IL-6. Some of these transcription factors may work together to enhance the expression of acute-phase SAA. For example, hepatocyte-specific mutations of binding sites for NF-κB and STAT3, but not either one alone, markedly reduce SAA1 expression by hepatocytes [66]. In one reported case, a mutation in the SAA1 promoter causes the sustained elevation of serum SAA1 level [59]. This T>C mutation leads to hereditary amyloidosis due to the increased production and systemic deposition of amyloid A. In addition to human SAA1, whose transcriptional regulation has been studied extensively, the transcriptional regulation of mouse *Saa3* has been reported. Saa3 is a functional protein in mice, and its distal regulatory element in the promoter functions as a transcription enhancer that contains three functional elements that interact with the CCAAT/enhancer-binding protein (C/EBP) and SAA3 enhancer factor (SEF) transcription factors [67,68]. These features can explain the rapidly induced tissue expression of Saa3 under inflammatory conditions.

The inducible feature of SAA expression has been used by several research groups to study the association and causal relationship of SAA expression and diseases and to develop therapeutic strategies. Zhang et al. reported in 2005 a human SAA1-luciferase transgenic mouse model that responded to inflammatory cues including sepsis, acute arthritis and contact hypersensitivity [69]. This work has been extended to Saa3, a non-hepatic inducible SAA protein in mice. A Saa3-luciferase reporter has been used for tracking the early development of renal fibrosis [70], for monitoring the development of diabetic kidney disease [71], and for the early detection of drug-induced nephrotoxicity [72]. In another study, the transcriptional activation mechanism of *Saa3* expression was used for the computational design of promoters for the targeted therapy of disease, in this case, rheumatoid arthritis [73,74].

## 3. Acute-Phase SAA in Microbial Infections

The serum concentration of SAA proteins increases markedly during acute infection [1]. As a result, SAA is a clinical biomarker for microbial infection. In several reported cases, SAA is also considered a prognosis marker for infectious diseases such as COVID-19 [75]. A related question is whether the elevated SAA concentration influences the spread, containment or eradication of the invading microbes. An early study indicates that SAA binds to outer membrane protein A of Gram-negative bacteria [11]. More recent studies have shown that recombinant human SAA1 and mouse Saa1 bind *C. albicans* [76]; and recombinant human SAA1 also binds *M. tuberculosis* [77]. The membrane association of acute-phase SAA plays a role for opsonization, thus facilitating the elimination of invading microbial pathogens [12,77,78].

In a study of skin infection by *S. aureus*, recombinant human SAA1 and SAA2 and mouse Saa1, Saa2 and Saa3 all bind bacterial membrane and exhibit a stronger bactericidal effect in lower pH conditions [79]. The same study also used gene KO mice to show that cutaneous infection was exacerbated when mouse *Saa1/Saa2* was deleted. Other gene knockout studies have been carried out to investigate the role for SAA in bacterial infection. Mice lacking *Saa3* displayed higher susceptibility to *P. aeruginosa*, a Gram-negative bacterium frequently found in hospital complications including ventilator-associated pneumonia [80]. Compared with wild-type mice, the *Saa3*^−/−^ mice showed pronounced neutrophil infiltration and a higher expression of TNF-α, KC/CXCL1 and MIP-2/CXCL2 in bronchoalveolar lavage fluid. The KO mice also had decreased superoxide production by neutrophils, resulting in the delayed clearance of invading *P. aeruginosa* and prolonged inflammation [80]. The SAA potentiation of NADPH oxidase activation was reported in this and another study [81]. Taken together, these findings indicate that SAA enhances host defense against microbial infection through several mechanisms.

Cheng and coworkers investigated an in vivo function of SAA using a transgenic approach. The investigators expressed human SAA1 in mice downstream of a scavenger receptor promoter [13]. The resulting transgenic mice (hSAA1-Tg) showed an expression of human SAA1 primarily in the lungs and at lower abundance in other tissues of the transgenic mice. In a systemic infection model of cecal ligation and puncture (CLP), the hSAA1-Tg mice exhibited resistance to infection and had lower mortality than the wild-type littermates [13]. In vitro experiments showed that SAA could bind LPS, the major component of endotoxin from Gram-negative bacteria. The SAA-LPS complex was endocytosed by CD68^+^ macrophages, effectively reducing serum LPS concentration by about 50%. Of interest, the formation and endocytosis of the SAA-LPS complex did not induce an elevated expression of inflammatory cytokines such as IL-1β and TNF-α. Instead, the tissue expression of TNF-α and IL-1β was reduced in the hSAA1-Tg mice compared with the wild-type mice that underwent the same infection procedure [13]. These results demonstrate the ability of acute-phase SAA1 to bind and neutralize LPS and to reduce its deleterious effect. These findings were confirmed in another study by Lv and coworkers who showed an SAA1-induced LPS internalization and blockade of the internalization by an SAA1 blocking peptide [82]. In a more recent study [83], the investigators used *Saa* TKO mice (triple knockout of the *mouse Saa1*, *Saa2* and *Saa3*) to demonstrate that these mice were more susceptible than the wild-type controls to bacterial infection as they succumbed to different ways of bacterial infections [83]. Altogether, there is clear evidence for an active role of acute-phase SAA proteins in host defense against microbial infection.

**Figure 1:**
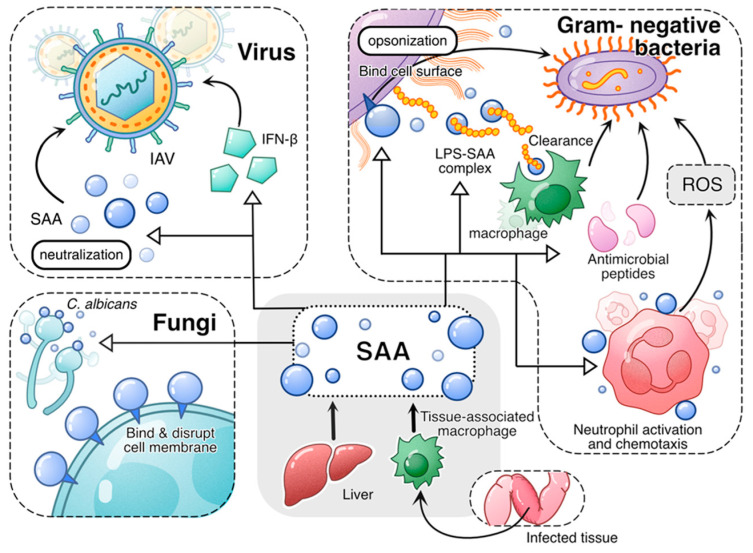
An overview of the antimicrobial activities of acute-phase SAA. Both liver-derived SAA and tissue-derived SAA play antimicrobial roles in infection. In viral infection, acute-phase SAA induces antiviral cytokines such as IFN-β and neutralizes virus, thus conveying antiviral activity. In fungal infection, acute-phase SAA binds and disrupts fungal cell membrane, thus killing the fungal cells. In infections caused by Gram-negative bacteria, acute-phase SAA employs multiple mechanisms to mitigate bacterial growth and spread. First, acute-phase SAA binding to bacterial cell wall serves as opsonin that promotes bacteria clearance by phagocytes. Secondly, acute-phase SAA binds and neutralizes LPS, facilitating its clearance by macrophages. Additionally, acute-phase SAA promotes expression of antimicrobial peptides. Finally, acute-phase SAA promotes phagocyte activation, leading to increased bactericidal activities including production of superoxide.

In addition to neutralizing bacterial products such as LPS, human SAA1 is reported to neutralize influenza A virus (IAV) through direct association [84]. Interestingly, only the SAA1 preparation from human blood, regardless of HDL association, has the neutralizing capability. The *E. coli*-produced recombinant SAA1 could not bind and neutralize IAV [84]. The SAA1-induced activation of interferon regulatory factor 7 (IRF7) [29,85] leads to the expression of IFN-β, which contributes to the anti-viral activities of SAA1 (avian SAA1 in this case) [86]. The antimicrobial activities of acute-phase SAA are schematically shown in Figure 1.

## 4. Acute-Phase SAA as a Proinflammatory Factor

**Figure 2:**
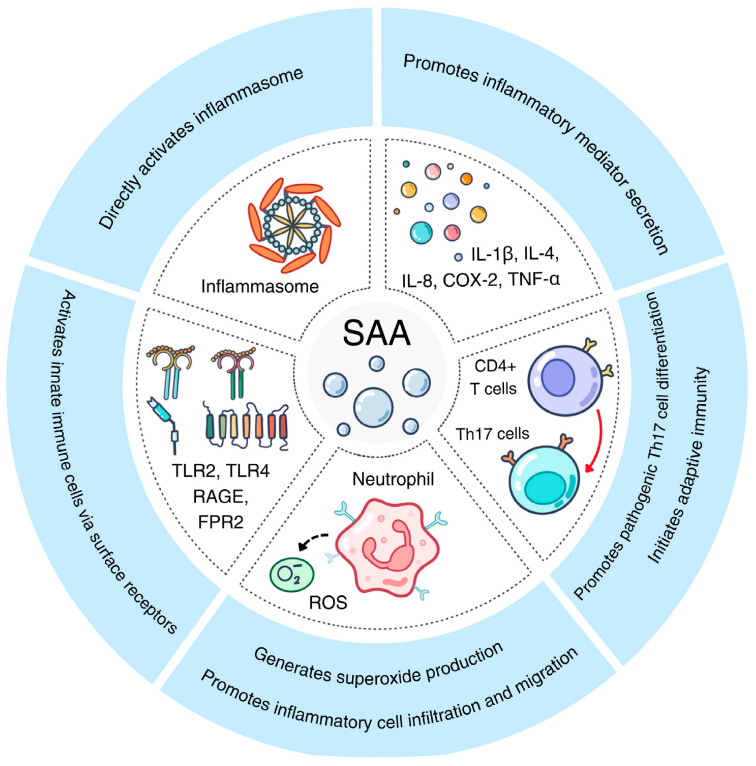
The proinflammatory activities of acute-phase SAA. Shown here are five pathways activated by acute-phase SAA through various receptors on innate immune cells, leading to Th17 differentiation, inflammasome activation and proinflammatory cytokine secretion.

Acute-phase SAA has long been associated with inflammation [1,3]. As shown in Table 1, a significant part of the SAA publications relate SAA with inflammatory disorders such as rheumatic arthritis [6] and inflammatory bowel disease [28]. These reports form the foundation for SAA being one of the major biomarkers of inflammatory diseases. The proinflammatory activities and underlying mechanisms are summarized in Figure 2.

In the past quarter century, the proinflammatory activities of acute-phase SAA have been extensively studied [87,88,89,90,91]. Early reports suggest that acute-phase SAA not only serves as an inflammatory biomarker but also actively participates in inflammation. The discovery that many of the identified receptors for acute-phase SAA, such as formyl peptide receptor 2 (FPR2) [92], Toll-like receptor 2 (TLR2) [93] and TLR4 [94], are established signal transducers in inflammatory cells further reinforces the notion that SAA acts at these receptors to induce inflammatory cytokine expression (summarized in [7]). Despite reports that contaminants in recombinant SAA preparation contribute to the observed proinflammatory activity, published studies using genetically altered mice demonstrate that a lack of Saa1/Saa2 or Saa3 reduces inflammation in disease models. Below are several examples.

### 4.1. Periodontitis

There was a report on Saa being a proinflammatory factor in periodontitis, which employed Saa gene knockout mice. In mice lacking *Saa1.1/Saa2.1*, there was reduced periapical inflammation [95]. Like these double-KO mice, *Saa3*^−/−^ mice displayed a redundant attenuation of periapical lesions. These findings provide support to the notion that Saa is proinflammatory in the mouse disease model.

### 4.2. Inflammation in the CNS

In a mouse model of cerebral ischemia, the deficiency of *Saa1/Saa2* in KO mice ameliorated ischemia-induced inflammation as shown in inflammasome activation [96]. Several SAA receptors including RAGE, TLR2 and TLR4 were suspected to participate in this phenomenon. In an AD mouse model, Saa3 was shown to inhibit astrocyte migration through the activation of p38 MAPK [97].

### 4.3. Priming Cells for Inflammatory Cytokine Release

In addition to inducing proinflammatory cytokine expression, SAA was reported to prime microglia for ATP-dependent IL-1β release [98]. In vascular smooth muscle cells, SAA1 was found to increase NOX4/ROS production to promote LPS-induced inflammation [81].

### 4.4. Th17 Differentiation of CD4^+^ T Cells

Recombinant human SAA1 was reported to induce a differential expression of IL-23 rather than IL-12 despite the sharing of a p40 subunit [91]. IL-17 is a downstream effector of IL-23, and recent work has shown that, in mice, Saa1/Saa2 promotes pathogenic Th17 cells, which in turn induces inflammatory cytokine expression [17].

There were several reports on the anti-inflammatory activities of SAA [8,10,13,80,83,99]. A careful analysis of the underlying mechanisms found that acute-phase SAA inhibits inflammatory response to infectious agents mainly through the enhancement of host defense and bactericidal activities, including superoxide production [80] and LPS neutralization [13], which help to eliminate pathogens and bacterial products causing tissue injury and inflammation. Moreover, a Saa3-dependent induction of IL-22 provides epithelial protection, thus reducing inflammatory injury to the infected tissues [10]. The established antibacterial activities of human SAA1, including binding to outer membrane protein A [11] and opsonizing the invading bacteria for phagocytosis [12], also facilitate the elimination of bacteria, thus promoting the resolution of inflammatory response to infection.

## 5. Acute-Phase SAA in Metabolic Regulation and Tumor Metastasis

Recent research has led to the identification of SAA involvement in metabolic regulation, suggesting a homeostatic role for SAA. Besides hepatic SAA produced in acute-phase response, a variety of cells can synthesize SAA protein that acts in a paracrine manner to influence the surrounding tissue environment. There are reports on SAA involvement in the formation of tumor microenvironment that favors metastasis. This section cites a number of reports published in recent years.

### 5.1. Metabolic Homeostasis

Mice with the targeted deletion of *Saa3* displayed a dysregulation of weight and altered immune response [100]. Notably, these KO mice developed adult-onset obesity and intrinsic airway hyperresponsiveness. CD4^+^ T cells in these mice showed impaired glycolytic activity, a decreased secretion of Th1 and Th2 cytokines and elevated IL-17A production when polyclonally stimulated. When infected with H1N1 influenza virus, these KO mice exhibited increased mortality, suggesting a homeostatic role for Saa3 in mice [100]. Saa1 expression may regulate bone density through the stimulation of osteoclast differentiation in mice [22]. This effect may be indirect and involves increased IL-17 production in mice with a hepatic expression of the human SAA1 transgene. Some of the effects of SAA on metabolic diseases could be achieved through the regulation of chronic inflammatory conditions [27]. Thus, SAA can also serve as a biomarker of these diseases.

### 5.2. Tumor Microenvironment

In a report published in *Nature*, Lee and coworkers showed that the hepatic expression of Saa1 and Saa2 was important to the formation of a pro-metastatic niche in mice [101]. This work relates hepatic Saa expression and elevated blood Saa levels (mainly Saa1 and Saa2) to the metastasis of cancer cells in the digestive system, which results from an Saa-induced local environmental change. Some cancer cells such as breast cancer also secrete SAA proteins, which is closely associated with the infiltration of neutrophils. Niu and coworkers reported an accumulation of suppressive granulocytes along with the progression of breast cancer in a TLR2-dependent manner in association with elevated human SAA1 protein production [102]. In terms of tumor metastasis, Saa3 in a mouse model has been shown to promote the lung metastasis of hepatocellular carcinoma through the formation of pre-metastatic niche, an action requiring IL-1β-induced Saa3 expression [103].

One mechanism employed by SAA in regulating cancer immunity is to predispose cancer cells, such as breast cancer cells, in an inflammatory tumor environment. This action may be explained by the ability of inflammatory factors such as IL-1β to induce NF-κB activation, which contributes to the induced expression of acute-phase SAA. The expressed acute-phase SAA then activates TLR1/TLR2 to induce an inflammatory tumor environment [26,104]. In cell-based studies, an overexpression of acute-phase SAA or the deletion of SAA genes are found to influence autophagy, with attenuated autophagy in human SAA1-overexpressed cells and enhanced autophagy with the deletion of the SAA genes from the cell lines [105,106]. In addition, adipocyte reprogramming associated with triple-negative breast cancer aggressiveness is influenced by human SAA1 through two of its receptors, CD36 and P2XR7 [107].

### 5.3. Macrophage Polarization

In addition to influencing cancer cell metastasis, SAA can exert its regulatory effect on tumor-associated macrophages that play important roles in the tumor microenvironment. One such reported case is colorectal cancer invasion that could be affected by tumor-derived IL-1β, which induces the expression of human SAA1. The expressed SAA1 then upregulates MMP-9 in macrophages to facilitate the invasion of cancer cells [108]. In a more recent report, Wu and coworkers observed an “invasive zone” in liver cancer in which damaged hepatocytes produce a large amount of acute-phase human SAA proteins to recruit macrophages and promote their M2 polarization [109]. As a result, there is an SAA-driven local immunosuppression that favors tumor progression.

**Figure 3:**
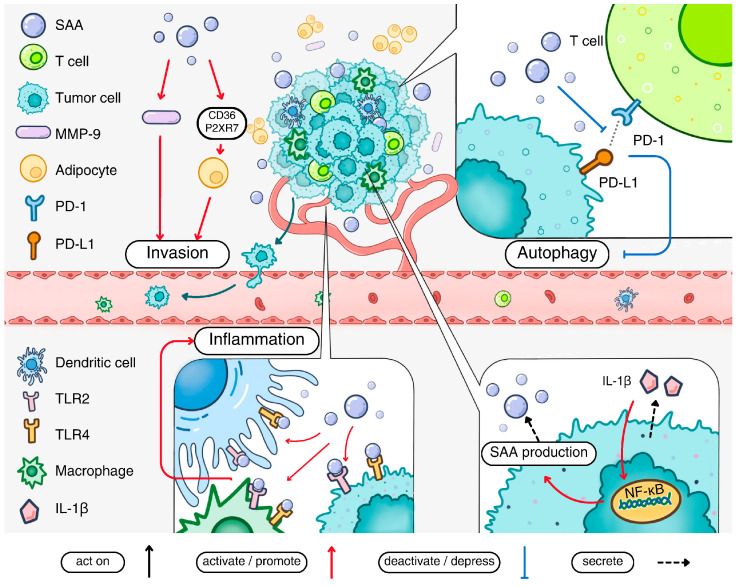
The pathological impact of acute-phase SAA in cancer. Acute-phase SAA production is stimulated by IL-1β present in the tumor microenvironment. The secreted acute-phase SAA activates cell surface receptors including TLR2 and TLR4 to promote the inflammatory tumor environment. Acute-phase SAA also promotes metastasis of cancer cells by inducing MMP-9 production. The invasion of cancer cells is facilitated by adipocyte reprogramming via the SAA-CD36-R2XR7 axis. Acute-phase SAA can inhibit the interaction between PD-1 and PD-L1, thereby suppressing autophagy. Finally, acute-phase SAA stimulates macrophage M2 polarization and contributes to a pro-metastatic niche in certain types of cancers.

Taken together, accumulating evidence supports a tumorigenic effect of acute-phase SAA. This is accomplished through a direct and indirect influence on tumor cells, on the tumor microenvironment, the formation of pre-metastatic niche and the suppression of anti-tumor immunity involving molecules such as α-PD-1 [110]. One example is that acute-phase Saa1 and Saa2 production in mouse liver serves to abrogate tumor surveillance by T cells, thus promoting tumorigenesis [111]. These and other mechanisms for acute-phase SAA participation in tumorigenesis are summarized in Figure 3.

## 6. Acute-Phase SAA as an Immunomodulator

Acute-phase SAA is generally considered a player in innate immunity. However, emerging evidence indicates the potential involvement of acute-phase SAA in macrophage polarization and T lymphocyte differentiation. Acute-phase SAA is also implicated in the induction of type 2 immune response [16,17,91,112].

### 6.1. Influence of Acute-Phase SAA on Macrophage Phenotype

Human SAA1 causes macrophage polarization, but the directions vary in different studies. Sun and coworkers reported an induction of M2 macrophage phenotype by recombinant SAA1, based on gene expression profiling and enhanced efferocytosis [85]. Macrophage recruitment and M2 polarization was also observed in the study of the “invasive zone” of liver cancer in which a subpopulation of damaged hepatocytes produces excessive SAA1 and SAA2 for local immunosuppression in human patients [109]. In contrast, Gaiser and coworkers found that Saa1 induces classically activated macrophages with M1-associated genes, which also enhances fibril formation in mice [113]. Lu and coworkers further illustrated the pathway through which Saa3 induces M1 macrophage differentiation, which could be negatively regulated by artesunate, an antimalaria drug [114]. The difference in macrophage phenotypes could be related to the experimental conditions including the SAA preparations used.

### 6.2. Type 2 Immune Response

There are reports that associate acute-phase SAA with type 2 immunity. Recombinant human SAA1 is reported to induce the ex vivo expression of IL-33 [115], an epithelial cell-derived alarmin and pleiotropic cytokine of the IL-1 family that promotes the expression of Th2-associated cytokines such as IL-4, IL-5 and IL-13 [116]. The biological consequence of this phenomenon was unclear until the publication of a recent report that illustrates an intricate mechanism linking house-dust-mite-associated allergy with SAA1 as a soluble pattern recognition receptor (sPRR) [112]. The group 13 mite allergens bind Saa1 on mucosal surface and break up the Saa1 hexamers into dimers and monomers, which are physiologically relevant agonists of Fpr2. This conversion is responsible for the induced expression of IL-33, leading to the production of Th2 cytokines including IL-4, IL-5 and IL-13, the latter triggering IgE production. This work demonstrates the pathophysiological presence of SAA proteins in different oligomeric states, some with receptor-activating capability and others not. The regulation of the quaternary structures of SAA through its conversion to biologically active forms is becoming a key factor for SAA activation and a potential target for therapeutic intervention [117].

### 6.3. Th17 Immunity

Acute-phase SAA is reported to play a role in intestinal immunity in part through the regulation of a network of genes. An example is vitamin-A-induced Saa1/Saa2 expression through epithelial retinoic acid receptor beta (RARβ), leading to IL-17 expression by intestinal Th17 cells [118]. This loop connects intestinal epithelial RARβ to the regulation of immunity by Th17 through mouse Saa1 and Saa2. In a series of studies conducted by Littman and coworkers, Saa1/Saa2 was shown to play a pivotal role in Th17 immunity using mouse models of inflammatory bowel disease and the colonization of segmented filamentous bacteria (SFB) [16,17,119]. In mice, SFB colonization induces Saa1/Saa2 expression in terminal ileum, and the induced Saa acts on lamina propria dendritic cells to promote Th17 cell differentiation [119]. Recombinant human SAA1 was also used in this study. The SAA1-induced Th17 immunity helps to prevent mucosal infection by other pathogenic bacteria. Mice express both Saa1/Saa2 and Saa3, and these Saa isotypes play different roles in Th17 immunity. While systemic Saa (Saa1 and Saa2) produced by hepatocytes promotes the differentiation of CD4^+^ T cells into Th17 cells, local SAA (Saa3 in this case) enhances the pathogenic effect of Th17 cells by promoting inflammation [17]. In a mouse model of SodA-induced sarcoidosis, recombinant human SAA1 serves to balance Th17/Treg in a CCL20-dependent manner, and the blocking of CCL20 could partially reverse the expression of Th17-related cytokines [120]. Taken together, the SAA promotion of Th17 immunity contributes to the proinflammatory activities of acute-phase SAA proteins.

## 7. SAA Isotypes and Functional Redundancy

**Figure 4:**
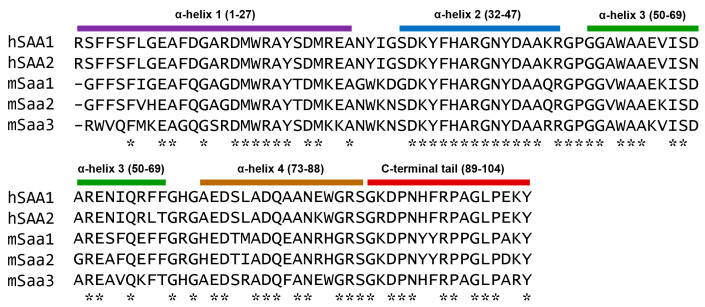
Comparison of the amino acid sequences of acute-phase human and mouse SAA proteins. Mature SAA protein sequences are shown, and identical amino acids are marked with asterisks (*) below the aligned sequences. Predicted α-helices, based on published structure of human SAA1 [18], are shown as color bars above the sequences with the beginning and ending amino acids numbered in parentheses.

Among the acute-phase SAA proteins, human SAA1 and SAA2 are hepatically produced and structurally similar (94% sequence identity). Likewise, mouse Saa1 and Saa2 are 93% identical in a primary sequence [30,121]. The similarity between human SAA1 and the three mouse acute-phase Saa proteins is lower, at 73–74% [121]. The aligned sequences of the human and mouse acute-phase SAA proteins are shown in Figure 4.

As shown in Figure 4, the human and mouse SAA proteins are highly conserved in α-helix 2 and the connecting segment between α-helix 2 and α-helix 3. The sequences are less conserved in α-helix 1 and the connecting segment between α-helix 1 and α-helix 2. In addition, Saa3 is less homologous than Saa2 to Saa1 (83% vs. 93%) [121]. These differences signal possible functional divergence, but the major difference in SAA proteins between human and mice lies in the functional expression of *Saa3* in mice and pseudogene in humans [122]. In genetically modified mouse models, the mouse *Saa1.1* and *Saa2.1* are often deleted together [99]. Mouse *Saa3* is functionally expressed as an inducible SAA protein [123] produced locally rather than hepatically. In several reported cases, *Saa3* KO mice displayed phenotype highly similar to that of the *Saa1.1/Saa2.1* DKO mice, suggesting that these mouse acute-phase Saa proteins may have essential and non-redundant functions in the models tested. For example, these DKO mice showed susceptibility to dextran sodium sulfate (DSS)-induced colitis, with compromised bactericidal activity in cultured epithelial cells [99]. A similar phenotype was observed in *Saa3* KO mice, with shortened colon length, increased bleeding and reduced body weight [10]. In the *Saa3* KO mice, there was a reduced expression of antimicrobial peptides and the epithelium-protecting cytokine IL-22 despite the presence of Saa1 and Saa2 [10]. These findings suggest that the presence of both *Saa1/Saa2* and *Saa3* is necessary for the homeostasis of normal colon functions, and the lack of either *Saa1/Saa2* or *Saa3* could lead to compromised integrity in colon epithelium and attenuated mucosal immunity.

In the *ApoE*^−/−^ mice for the induction of atherosclerosis, the over-expression of Saa3 using the adeno-associated virus led to a marked increase in the atherosclerosis lesion area compared to the control mice, and the suppression of *Saa3* expression decreased atherosclerosis in mice with the genetic deletion of *Saa1.1* and *Saa2.1* [124]. The augmentation effect was obvious in this mouse model of atherosclerosis, leading to the conclusion that Saa3 is pro-atherogenic.

The study conducted by Lee and coworkers [17] provides valuable information that distinguishes the functions of SAA isotypes in Th17 immunity. This work demonstrates that Th17 differentiation is mostly influenced by hepatic Saa1 and Saa2, but the gut-produced Saa3 potentiates the pathogenicity of Th17 cells. Therefore, different SAA isotypes may have different functions in a context (tissue)-specific manner.

## 8. Studies of SAA Using Genetically Modified Mice

Genetically modified mice are widely used to elucidate the functions of SAA in vivo. This approach is often combined with the pathogenic characterization of mouse models of human diseases, which not only validates in vitro findings of SAA functions but also unveils new roles of SAA in the homeostatic regulation of immunity and metabolism. The Saa knockout (KO) and transgenic (Tg) mice also provide direct evidence for the proinflammatory and pro-metastatic functions of Saa in selected disease models. To date, mice with individual or combined deletions of Saa genes have been generated, including the *Saa3* KO, *Saa1.1/Saa2.1* double-knockout (DKO), *Saa1.1/Saa2.1/Saa3* triple-knockout (TKO) and *Saa1.1/Saa2.1/Saa3/Saa4* quadruple-knockout (QKO) mice. Moreover, the transgenic expression of human SAA1 has been accomplished in mice, using promoters that convey selectivity in different tissues.

### 8.1. Host Defense

Mice lacking *Saa1.1/Saa2.1* were more susceptible to bacterial infection than the wild-type controls with aggravated damage to colonic epithelium [99]. A similar phenotype was observed in *Saa3* KO mice when challenged with dextran sulfate sodium (DSS), and the supplement of Saa3 protein to the KO mice partially relieved the symptoms [10]. In mice infected with *P. aeruginosa*, the *Saa3* KO genetic background displayed more severe and longer infection than the wild-type littermates. Phagocytes in the KO mice showed less effective bacterial elimination in part due to weaker superoxide production [80]. These findings provide in vivo evidence for the antimicrobial activities of Saa that was initially observed ex vivo. In mice, acute-phase Saa proteins are found in colonic mucosa and airway epithelium for the enhancement of local immunity at the mucosal surface in part through the induced expression of antibacterial peptides including Reg3β and Reg3γ and the epithelium-protecting cytokine IL-22 [10]. In *Saa* TKO mice, infection with CLP, cecal slurry or LPS injection induced more severe kidney and lung injury than in wild-type controls, with significantly increased mortality [83]. These results are consistent with previous findings in human SAA1 Tg mice that displayed enhanced protection against injuries caused by CLP and LPS administration, mainly through the increased ability of human SAA1 to promote LPS clearance by macrophages [93]. In a cutaneous *S. aureus* infection model, the Saa DKO mice displayed larger areas of infection than the wild-type controls [79]. These in vivo studies support an antibacterial function of SAA proteins.

### 8.2. Modulation of Inflammation in Central and Peripheral Nervous System

Work carried out in several laboratories has generated different results regarding an SAA role in the nervous system. In an AD model using APP/PS1 mice with a *Saa3* KO background, the absence of Saa3 led to increased tau protein hyperphosphorylation in response to systemic LPS administration. The administration of recombinant SAA1 protein reduced tau hyperphosphorylation while activating microglial cells [9]. In another study using the APP/PS1 mouse model, *Saa3* deficiency exacerbated astrocyte activation and increased the number of astrocytes around Aβ deposits [97]. In a study using the *Saa1.1/2.1* DKO mice, the administration of combined Saa proteins (mouse Saa1, Saa2 and Saa3) induced a microglial activation of inflammasome through the mouse RAGE, TLR2 and TLR4 [96]. Finally, SAA may be involved in peripheral nervous system as shown in a study that used a curli-challenged primary murine myenteric network model. In that model, *Saa3* deficiency attenuated curli-induced DNA damage and replication [125]. These findings indicate that the CNS and PNS effects of SAA are related to its ability to regulate local inflammatory response.

### 8.3. Induction of Th17 Immunity

Accumulating evidence indicates that SAA induces Th17 immunity, which in turn promotes mucosal defense and autoimmunity. As mentioned above, the work by Sano and coworkers showed that SFB could potentiate autoimmunity in mice through SAA-dependent IL-17A production by RORγ(+) T cells in the ileum [16]. Lee et al. extended this work by demonstrating the ability of hepatic Saa (encoded by *Saa1.1/Saa2.1*) to induce IL-17 immunity and local Saa (encoded by *Saa3*) to aggravate a pathogenic effect of the Th17 response [17]. In another study using the transgenic expression of hepatic human SAA1, IL-17 was shown to be upregulated in γδ T cells by hepatic hSAA1 through TLR2 [21]. In addition to commensals such as SFB, pathogens including *S. enterica* can induce IL-17 production, which is related to vitamin A-dependent intestinal immunity [118]. The mechanism underlying the SAA induction of Th17 immunity has not been fully delineated, but it has been shown that, in monocytes, recombinant SAA1 stimulates IL-23 expression, which then induced IL-17 expression [91]. In mice, this process also involves the IL23R/IL-22 circuit [16]. Further understanding of how SAA produced systemically or locally regulates Th17 immunity may help to appreciate how SAA bridges innate immunity and adaptive immunity.

### 8.4. SAA and the Development of Type 2 Immunity

Type 2 immunity is characterized by the production of IL-4, IL-5 and IL-13, often in response to infections and allergens. House dust mite induces allergic response with characteristics of type 2 immunity. In a recent report employing *Saa1.1/Saa2.1* DKO mice, Smole and coworkers found a critical role for SAA in the mite-induced allergic response [112]. SAA1 interacts directly with the mite fatty acid-binding proteins (FABPs) that promote pulmonary type 2 immunity. In this case, SAA1 binding to the mite FABPs (Der p 13 and Blo t 13) changes its quaternary structure from non-functional hexamers to functional dimers and monomers, which then activates FPR2 and elicits an IL-33-dependent production of IL-4, IL-5 and IL-13. This work not only links acute-phase SAA to type 2 immunity but also illustrates a mechanism by which SAA activity is regulated at the level of higher-order structures.

### 8.5. SAA in Tumor Metastasis

Mouse models have a clear advantage in studies of SAA in metabolism and cancer. As mentioned in Section 4.2, SAA in mouse liver has been shown to promote the formation of a pro-metastatic niche, a finding confirmed with the use of *Saa1.1/Saa2.1* DKO mice [101]. The local administration of Saa3 also promotes the lung metastasis of hepatocellular carcinoma through the formation of pre-metastatic niche [103]. There is also a link between elevated SAA level and the accumulation of tumor-associated suppressive granulocytes [102].

There have been increasing numbers of publications using genetically modified mice in SAA research, which provides direct evidence for in vivo functions of SAA. Table 2 is a brief summary of results from studies using SAA KO and Tg mice.

## 9. Variations in SAA Preparations and Receptor-Dependent Signaling

Conflicting results have been obtained from SAA studies, creating confusion in the understanding of SAA signaling mechanisms. A recent survey of SAA literature found that SAA expressed from different sources or administered through different routes has a profound impact on experimental outcome. The commercially available recombinant human apo-SAA with two amino acid substitutions of the human SAA1.1 sequence was used in most of the in vitro studies published to date, showing strong inflammatory response in various cell models. The apo-SAA preparation was also used in studies resulting in the initial identification of SAA receptors (summarized in [7]). It was later realized that the recombinant human apo-SAA differs in primary sequence from the wild-type human SAA1, with substitutions at positions 61 and 72 and an addition of methionine at its N-terminus. These modifications may have contributed to the strong biological activities and proinflammatory properties of the SAA preparation [15,133]. Struyf and coworkers recently reported that bacterial contaminants in the apo-SAA preparation could contribute to an SAA activation of TLR2 and TLR4. They showed that purification procedures that remove the contaminants generated an SAA preparation that lacked the TLR2- and TLR4-stimulating activity while retaining the FPR2-activating effect [134]. These observations raise important questions on whether the proinflammatory effects observed in vitro accurately reflect the SAA functions in vivo. The following discussions are provided in an attempt to identify potential causes for the discrepancies reported in the published literature, with an emphasis on FPR2, TLR2 and TLR4.

### 9.1. Agonistic Activity of SAA at FPR2

In 1994, Badolado and coworkers reported that recombinant human apo-SAA could induce calcium mobilization in neutrophils [87]. Su et al. followed by reporting in 1999 that human FPR2 could be activated by recombinant human apo-SAA to induce calcium mobilization and chemotaxis at micromolar concentrations [92]. Subsequently, He and coworkers found that recombinant human apo-SAA could stimulate neutrophils to produce IL-8, an activity mediated by FPR2 [90]. FPR2 is a G-protein-coupled chemoattractant receptor that is homologous to FPR1 in sequence, and FPR2 signaling leads to degranulation and superoxide production as well as chemotaxis of neutrophils [135]. Therefore, functions that were later reported, including the reduction in superoxide generation associated with a compromised clearance of *P. aeruginosa* in *Saa3*^−/−^ mice [121], could be attributed to SAA binding to and the activation of FPR2. These findings were extended from humans to mice in which recombinant apo-SAA was found to act through the mouse equivalent of human FPR2 [136]. The chemotactic activity of recombinant human SAA plays an important role in wound healing through FPR2-dependent epithelial migration [137].

Several other studies have attributed their observed biological activities of SAA to FPR2. For example, house dust mites induce allergic responses in part through Saa1-induced FPR2 activation that leads to IL-33 expression and type 2 immunity in mice [112]. The repurification of recombinant apo-SAA did not affect the chemotactic activity of SAA, which is mediated by FPR2 [134]. All these studies support an agonistic activity of acute-phase SAA at FPR2, which is partially responsible for the proinflammatory effects of SAA [90,138]. It should be noted that FPR2 is not sensitive to LPS or bacterial lipoproteins in the SAA preparations; hence, the agonistic activity of SAA at FPR2 is consistently observed with SAA preparations from *E. coli* expression system with or without repurification. Interestingly, the transgenic expression of human SAA1 in mouse lungs did not lead to increased neutrophil infiltration; in contrast, there were less neutrophils in the infected lungs of the transgenic mice than the wild-type controls undergoing CLP [13]. This observation may be explained by recent findings that factors influencing the quaternary structure of SAA can play a role in the regulation of SAA agonism in vivo [112,117].

### 9.2. SAA Activation of TLR2 and TLR4

These receptors were originally identified for the detection of bacterial products including lipoproteins, teichoic acid and LPS [139]. As a result, TLR2 and TLR4 are pattern recognition receptors (PRRs). LPS has been shown to bind tightly to SAA [13]. It is, therefore, not unexpected that SAA preparations from the *E. coli* expression system can be contaminated with bacterial products that may contribute to the biological activities through TLR2 and TLR4. Supporting this notion, Burgess and coworkers reported that SAA from a commercial source activates TLR2 because of bacterial contaminants. The authors also showed that their SAA preparation from mammalian cells did not activate TLR2 for proinflammatory cytokine secretion or Th17 differentiation [14].

It is technically feasible to purify *E. coli*-derived recombinant SAA to near homogeneity for crystallization [18]. With the commercially available apo-SAA, the LPS concentration was controlled to a very low level (<1 EU/μg protein), and LPS at such a low concentration cannot induce the response elicited by recombinant apo-SAA. In one of the published studies, the SAA preparation was boiled for 20 min to denature SAA protein but retain LPS activity, and such a treatment diminished the biological activity of recombinant Apo-SAA [91]. It is suggested that the contaminating LPS alone may not be sufficient to induce the observed proinflammatory effects unless it is bound to SAA. However, the SAA-LPS complex characterized in another study did not display proinflammatory activities at bone-marrow-derived mouse macrophages, which express TLR4. In fact, the complex was removed by macrophages without eliciting proinflammatory cytokine production [13].

Studies using gene knockout mice and transgenic mice have helped to investigate the in vivo functions of SAA without relying solely on recombinant SAA preparations. Several studies also combined Saa gene knockout with *Tlr4* knockout. In two published studies, it was shown that TLR4 mediated the proinflammatory activity of Saa in mice, and such activities were diminished in *Tlr4* knockout mice in studies of periodontal inflammation [95] and hepatic steatosis [128]. These findings suggest an in vivo role for TLR4 in mediating the biological activities of SAA.

In addition to TLR4 signaling, there is evidence for or against a role of TLR2 in SAA signaling. For example, the original report on TLR2 being an SAA receptor had included a control human SAA1 produced by mammalian cells (CHO) grown in serum-free medium, which also induced TLR2 signaling [93]. Several in vivo studies have shown an involvement of TLR2 in SAA signaling. Choi and coworkers studied transgenic mice with a hepatic expression of human SAA1 and found that SAA1-induced IL-17 expression by γδ T cells was subject to inhibition by a TLR2 inhibitor, CU-CPT22 [21]. This result, using an in vivo expression of transgenic SAA1 devoid of bacterial contamination during protein expression and purification supports the involvement of TLR2 in mediating the proinflammatory action of SAA. In a recent study, Li and coworkers reported that a viral vector-mediated hepatic expression of Saa1 could promote synovial macrophage activation through NFAT5 [140]. Since the NFAT5 pathway could be activated through upstream receptors including TLR2 and TLR4, the authors generated *Tlr2/Tlr4* DKO mice and convincingly showed the dependence of the Saa1 effect on TLR2 and TLR4. The in vivo evidence from these studies is consistent with early reports on the participation of TLR2 and TLR4 in SAA signaling [93,94].

### 9.3. Higher-Order Structure of SAA and Its Bioactivity

SAA is a heteromorphous protein with propensity of aggregation as seen in all amyloid proteins. It is, therefore, possible that SAA proteins expressed in different systems have different tertiary or quaternary structures, which subsequently display different biological functions. There are published studies on crystal structures of full-length SAA. The structure of human apo-SAA1 reported in 2014 is present in monomer, dimers and hexamers (each hexamer formed by two identical trimers), with the protomer having 4 transmembrane helices [18]. The retinol-bound Saa3 displays a similar structure but associated as a tetramer [19]. In addition, the crystal structure of SAA2-55 was resolved, and it showed a different structure with fibril packing. Furthermore, a molecular dynamics simulation demonstrated that acidic conditions favor fibril formation [141]. Protein folding can be influenced by many factors that influence the bioactivity of SAA. Additionally, newly released SAA during acute-phase response rapidly associates with HDL in blood circulation [142]. The SAA proteins expressed in inflammatory tissues may aggregate through intermolecular interactions involving certain regions of SAA [25], and these factors must be considered in evaluating experimental results obtained from SAA studies. SAA protein encoded by a particular SAA gene may display distinct properties dependent on protein folding and association with another molecule. A recent study showed that, at neutral pH, lipid-free SAA is intrinsically disordered and rapidly cleared from circulation [143]. The association of acute-phase SAA with HDL prevents the formation of SAA fibrils and promotes the SAA encapsulation of lipids into nanoparticles [144]. SAA monomer has a concave hydrophobic surface and a hydrophilic interior partially filled with water [18,19,131]. This structure makes it easier for misfolding unless it is stabilized through the association of its hydrophobic surface with lipids [145]. In the four-helix structure of SAA monomer, h2 and h3 with the connecting sequence of GPGG motif are critical to the curvature of the hydrophobic surface [25,146].

The work by Smole et al. provides clear evidence that the higher-order structure of SAA is subject to regulation by environmental factors, in this case, the mite Fatty Acid Binding Protein (FABP) could induce the transformation of SAA hexamers to dimers and monomers, thereby activating FPR2 for type 2 immune response [112]. In another published report, it was shown that SAA assembly is required for its proinflammatory response [147]. Based on the discovery of a 5-amino acid peptide from arthritis patients, Hemed-Shaked et al. reported that the CD44-variant-derived sequence of the peptide can associate with SAA protein and prevent its assembly. They attributed the anti-inflammatory effect of this interaction to the prevention of SAA from assembly [147]. Moreover, SAA could form a complex with citrullinated fibrinogen, and this complex could cause vascular metastagenesis [148].

SAA also exhibits a detergent-like property, disrupting anionic liposomes by the spontaneous formation of small vesicles or micelles [25]. This action is pH-dependent and occurs under acidic conditions [79]. Therefore, SAA can directly bind to bacterial cell surface and disrupting the cell membrane in acidic conditions.

Taken together, the various and sometimes inconsistent results obtained from SAA studies may be attributed to the presence of SAA in different forms, either as homo-oligomers (e.g., hexamers) or in association with another factor (e.g., HDL). It is possible that different protein expression systems may influence SAA folding that lead to inconsistent results.

## 10. Conclusions and Future Perspectives

Studies in the past 20 years have clearly demonstrated biological activities of acute-phase SAA both in vitro and in vivo. These activities of SAA may influence pathophysiological processes including inflammation and tumor metastasis. More importantly, SAA has strong activities against microbial infection, which justifies its production in acute-phase response in large amounts. There are, however, knowledge gaps in our understanding of how SAA works in vivo. There is still a belief that many of the reported bioactivities of SAA are the results of contaminants in recombinant SAA preparations. The current literature does not provide sufficient evidence for a causal relationship between elevated SAA expression and the associated diseases. Meanwhile, research on SAA multimers has begun to shed light on a regulatory mechanism for SAA activities in vivo. With the adaptation of research tools including genetically modified mice, additional functions of SAA are bound to be unveiled so will be the underlying mechanisms. The regulation of SAA bioactivity at the level of quaternary structure by hexamer-to-dimer/monomer transformation points to a new direction for SAA research. These and other advancements in SAA studies are expected to accelerate the pace of discovery and application for better diagnosis and treatment of inflammatory, metabolic and malignant diseases.

## Figures and Tables

**Table 1:** Association of SAA expression with major diseases.

Disease Type	Clinical Role	SAA-Related Findings	Ref.
**Infectious disease**	Diagnostic biomarker	Elevated levels in bacterial or viral infection (e.g., sepsis, pneumonia, tuberculosis and COVID-19) and chronic infection (e.g., chronic hepatitis).	[34,35,36,37,38,39]
	Progression of infection	Higher levels correlate with severity and duration of infection.	[36]
	Prognostic biomarker	Sustained SAA expression marks poor prognosis with low survival rate in COVID-19 patients.	[40]
**Inflammatory disease**	Diagnostic biomarker	Markedly increased expression in rheumatoid arthritis, systemic lupus erythematosus and inflammatory bowel disease.	[41,42,43,44]
**Cardiovascular disease**	Diagnostic biomarker	Elevated levels in cardiovascular diseases; predicts cardiovascular risk.	[32,45,46,47]
	Atherogenesis	A contributing factor of atherogenesis through involvement in cholesterol metabolism, participation in detoxification, depression of immune responses and interference with platelet functions.	[48,49]
	Prognostic biomarker	Higher expression levels predict a poor prognosis.	[46,47,50]
**Cancer**	Diagnostic biomarker	Elevated levels in breast cancer, lung cancer, liver cancer, ovarian cancer and pancreatic cancer.	[51,52,53,54]
	Cancer progression	Higher expression levels support tumor metastasis by modulating tumor environment; *SAA1.5/1.5* genotype predisposes individual to nasopharyngeal carcinoma.	[33,54,55]
	Prognostic biomarker	Elevated levels correlate with a shorter overall survival in different types of cancer.	[53,56]
**Amyloidosis**	Diagnostic biomarker	Elevated levels in amyloidosis in major organs such as the kidney.	[57,58,59]
**Metabolic disease**	Obesity and metabolic syndrome	Increased expression found in obesity. SAA and leptin expression have a strong connection in adipose tissue.	[60,61]
	Type 2 diabetes	Elevated levels in type 2 diabetes.	[62]
**Other disease**	Diagnostic biomarker	Higher expression levels in trauma, surgery and organ transplant rejection.	[35,63]

**Table 2:** SAA knockout and transgenic mice and their phenotypes.

Mouse Strain	Disease Model	Summary of Results	Ref.
**DKO** **(*Saa1/Saa2*)**	DSS-induced colitis	*Saa1.1/Saa2.1* DKO led to loss of protection against acute injury to colon epithelium.	[99]
		*Saa1.1/Saa2.1* DKO exacerbated inflammation-associated damage and tumorigenesis of colitis-associated cancer.	[126]
	Acute SFB colonization	*Saa1.1/Saa2.1* DKO abrogated IL-17A induction in Th17 cells.	[16]
	Cutaneous bacterial infection	*Saa1.1/Saa2.1* DKO and combined administration of mouse Saa1/Saa2/Saa3 suggested antibacterial activity against cutaneous bacterial infections.	[79]
	*H. Hepaticus* infection	A study using *Saa1.1/Saa2.1* DKO, *Saa1.1/Saa2.1/Saa3* TKO and hepatic *Saa1* transgenic mice, combined with administration of recombinant human SAA1 and mouse Saa1, led to the conclusion that SAA induces pathogenic Th17 cells and promotes inflammatory disease.	[17]
	Mouse periapical lesions	Using *Saa1.1/Saa2.1* DKO and *Saa3* KO mice, combined with administration of recombinant human SAA1, this study concluded that the SAA proteins act through TLR2 and TLR4 to promote periapical inflammation. This conclusion was verified in gene knockout mice lacking TLR2 and TLR4.	[95]
	Insulin resistance	Viral vector-mediated silencing of *Saa1* led to insulin resistance.	[127]
	Fatty liver disease	Combined use of *Saa1.1/Saa2.1* DKO, Tlr4 KO and AAV8-mediated hepatic expression of Saa1 led to the conclusion that hepatic *Saa* exacerbates steatosis via TLR4-mediated NF-κB signaling pathway.	[128]
	Cerebral ischemia and reperfusion	*Saa1.1/Saa2.1* DKO and combined administration of mouse Saa1/Saa2/Saa3 led the authors to conclude that Saa exacerbates the symptoms of cerebral ischemia and reperfusion injury. SAA signaling plays a critical role in regulating NLRP3-induced inflammation and glial activation in the ischemic brain.	[96]
	HDM allergic response	Combined use of *Saa1.1/Saa2.1* DKO and forced expression of Saa1 led to the finding that SAA initiates type 2 immunity at mucosal surfaces through the SAA1-FPR2-IL-33 axis. The results were verified in cell-based assays.	[112]
	*S. enterica* infection	Using *Saa1.1/Saa2.1* DKO mice, the authors found intestinal epithelial Saa1/Saa2 could regulate Th17 cell effector function against *S. enterica* infection in a retinoid acid receptor B-dependent manner.	[118]
***Saa3*** **KO**	DSS-induced colitis	Combined use of *Saa3* KO mice and administration of recombinant Saa3 led to the finding that Saa3 protects colon epithelium against acute injury via IL-22.	[10]
	Sepsis (LPS injection)	Combined use of *Saa3* KO mice and administration of recombinant SAA1 led the authors to identify Saa3 suppression of LPS-induced tau hyperphosphorylation	[9]
	LPS treatment to BMDC	*Saa3* KO changes immunometabolic function and promotes adult-onset weight gain and dyslipidemia.	[129]
	Mouse periapical lesions	Saa promotes mouse periapical inflammation in both *Saa1.1/Saa2.1* DKO and *Saa3* KO mice.	[95]
	*P. aeruginosa* infection	*Saa3* KO mice were more susceptible to *P. aeruginosa* infection with more severe lung injury.	[80]
	AD (aged APP/PS1)	*Saa3* KO increased astrocyte migration in a p38 MAPK-dependent manner.	[97]
	Acute pancreatitis	Combined use of *Saa3* KO mice and administration of recombinant Saa3 protein found that Saa3 could enhance acute pancreatitis by inducing an RIP3-dependent necroptosis pathway in acinar cells.	[130]
	Bacterial amyloid curli-induced proinflammatory response	Combined use of *Saa3* KO mice and administration of recombinant Saa3 led to the finding that SAA3 fuels a feed-forward inflammatory response to the bacterial amyloid curli in the enteric nervous system.	[125]
**TKO** **(*Saa1/Saa2/*** ***Saa3*)**	*ApoE*^−/−^ mice	Comparison of *Saa1.1/Saa2.1* DKO with *Saa1.1/Saa2.1/Saa3* TKO mice in *ApoE*^−/−^ background led to the conclusion that Saa3, but not Saa1/Saa2, is relevant to progression of atherosclerosis.	[124]
	Sepsis mouse models	The *Saa1.1/Saa2.1/Saa3* TKO mice showed higher susceptibility to bacterial infection and LPS administration with more severe symptoms.	[83]
**QKO (*Saa1.1/Saa*** ***2.1/Saa3/*** ***Saa4*)**	*S. typhimurium* Infection	The QKO mice were used in a study of retinol binding to Saa3 in relation to Salmonella infection. It was found that QKO compromised adaptive immunity to *S. typhimurium* infection.	[131,132]
***SAA1*** **Transgenic**	Cecal ligation puncture for acute lung injury	Transgenic expression of human SAA1 provided enhanced protection of mice against CLP-induced sepsis and LPS-induced acute lung injury, through SAA1 binding to LPS and promotion of LPS clearance by macrophages.	[13]
	No treatment	SAA1 relates to psoriasis-like symptoms. Transgenic expression of human SAA1 increased IL-17-producing innate immune cells and decreased bone density.	[21,22]
